# Supplementary material for: Reconciling Mining with the Conservation of Cave Biodiversity: A Quantitative Baseline to Help Establish Conservation Priorities
Source: PLoS One. 2016 Dec 20;11(12):e0168348. doi: 10.1371/journal.pone.0168348 (PMC5173368; doi:10.1371/journal.pone.0168348)
Supplement: S1 Dataset — (ZIP) [file pone.0168348.s002.zip › Taxa/Serra Norte/SN_2013/N4WS/Lista N4WS-46.pdf]

CAVIDADE N4WS-0046

Classe	Ordem	Fam/Outros	Gên/Outros	Espécie	S-U	U-S	Zon.
Annelida	Oligochaeta			sp.		2	E
Arachnida	Acari	Erythraeidae		sp.2	X		P
Arachnida	Acari	Ixodidae	*Amblyomma*	sp.1	X		P
Arachnida	Acari	Opilioacaridae	*Neoacarus*	sp.1	X		P
Arachnida	Acari	Rhagidiidae		sp.1	X		P
Arachnida	Amblypygi	Phrynidae	*Heterophrynus*	*longicornis*	1		P
Arachnida	Araneae	Pholcidae	*Mesabolivar*	*eberhard*		X	E
Arachnida	Araneae	Salticidae	*Freya*	sp.1		X	E
Arachnida	Araneae	Salticidae	*Soesilarishius*	sp.1	X		P
Arachnida	Araneae	Theraphosidae		jovem		1	E
Arachnida	Araneae	Theridiosomatidae	*Plato*	sp.1	X	X	E
Arachnida	Araneae	Trechaleidae		jovem	1		P
Arachnida	Araneae	Uloboridae	*Uloborus*	sp.1		X	E
Arachnida	Pseudoscorpiones	Bochicidae		jovem	X		P
Arachnida	Pseudoscorpiones	Chernetidae	*Spelaeochernes*	sp.1		X	E
Arachnida	Pseudoscorpiones	Chthoniidae	*Pseudochthonius*	sp.1	X	X	E/P
Chilopoda	Geophilomorpha	Ballophilidae	*Ityphilus*	sp.1	1	1	E
Chilopoda	Scolopendromorpha	Scolopocryptopidae	*Tidops*	sp.1		1	E
Entognatha	Collembola	Paronellidae		sp.1	X		P
Entognatha	Diplura	Campodeidae		sp.1		X	E
Insecta	Blattodea			jovem	1		P
Insecta	Coleoptera	Staphylinidae	Pselaphinae	sp.41	X		P
Insecta	Coleoptera	Staphylinidae		sp.35	X		P
Insecta	Diptera	Ceratopogonidae		jovem	X		P
Insecta	Diptera	Limoniidae		sp.	X		P
Insecta	Diptera	Muscidae		jovem	X		P
Insecta	Diptera	Phoridae	Metopininae	sp.	X		P
Insecta	Diptera	Psychodidae	*Sciopemyia*	*sordellii*	X	X	E/P
Insecta	Heteroptera	Reduviidae	Reduviinae	jovem	1		P
Insecta	Hymenoptera	Formicidae	*Brachymyrmex*	sp.1	X		P
Insecta	Hymenoptera	Formicidae	*Nylanderia*	sp.3		X	E
Insecta	Hymenoptera	Formicidae	*Solenopsis*	sp.1	X		P
Insecta	Lepidoptera			jovem	X	X	E/P
Insecta	Orthoptera	Phalangopsidae	*Paraclodes*	sp.	3		E
Insecta	Orthoptera	Phalangopsidae	*Phalangopsis*	sp.	11	14	P
Insecta	Psocoptera	Psocomorpha		jovem		X	E
Amphibia	Anura	Dendrobatidae	*Epipedobates*	sp.	1		P
Amphibia	Anura	Strabomantidae	*Pristimantis*	cf. *fenestratus*	2	1	E
